# Flow and heat transfer in a Maxwell liquid film over an unsteady stretching sheet in a porous medium with radiation

**DOI:** 10.1186/s40064-016-2655-x

**Published:** 2016-07-12

**Authors:** Shimaa E. Waheed

**Affiliations:** Department of Mathematics, Faculty of Science, Benha University, Banha, 13518 Egypt; Department of Mathematics and Statistic, Faculty of Science, Taif University, Taif, Kingdom of Saudi Arabia

**Keywords:** Maxwell fluid, Liquid film, Thermal radiation, Unsteady stretching sheet, Porous medium

## Abstract

A problem of flow and heat transfer in a non-Newtonian Maxwell liquid film over an unsteady stretching sheet embedded in a porous medium in the presence of a thermal radiation is investigated. The unsteady boundary layer equations describing the problem are transformed to a system of non-linear ordinary differential equations which is solved numerically using the shooting method. The effects of various parameters like the Darcy parameter, the radiation parameter, the Deborah number and the Prandtl number on the flow and temperature profiles as well as on the local skin-friction coefficient and the local Nusselt number are presented and discussed. It is observed that increasing values of the Darcy parameter and the Deborah number cause an increase of the local skin-friction coefficient values and decrease in the values of the local Nusselt number. Also, it is noticed that the local Nusselt number increases as the Prandtl number increases and it decreases with increasing the radiation parameter. However, it is found that the free surface temperature increases by increasing the Darcy parameter, the radiation parameter and the Deborah number whereas it decreases by increasing the Prandtl number.

## Background

The flow and heat transfer within a thin liquid film due to the stretching surface in otherwise quiescent fluid are important because of their wide applications in a number of industrial engineering processes. Examples may be found in the cooling of a large metallic plate in a cooling path, design of various heat exchangers, wire and fiber coating, manufacturing plastic films, continuous casting, crystal growing,artificial fiber, reactor fluidization, chemical processing equipment, a polymer sheet, polymer extrusion, annealing and tinning of copper wires, etc. The flow problem within a liquid film of Newtonian fluid on an unsteady stretching surface where the similarity transformation was used to transform the governing partial differential equations describing the problem to a non-linear ordinary differential equation with an unsteadiness parameter first are studied by Wang (1990). Many authors (Usha and Sridharan 1995; Andersson et al. 2000; Dandapat et al. 2003, 2007; Wang 2006; Dandapat and Maity 2006; Liu and Andersson 2008; Noor and Hashim 2010; Mahmoud 2010; Ray and Mazumder 2001; Abel et al. 2009) investigated thin liquid film under different situations.

Many important fluids, however, such as molten plastics, polymers, etc., are non-Newtonian in their flow characteristics. The flow of non-Newtonian fluids are finding increasing applications in several manufacturing processes. Flow of a thin liquid film of a power-law fluid caused by the unsteady stretching of a surface studied numerically by Andersson et al. (1996) and analytically by Wang and Pop (2006). The thin film flow problem with a third grade fluid on an inclined plane hase beed investigated by Siddiqui et al. (2008). Chen (2007) examined the effect of Marangoni convection of the flow and heat transfer within a power-law liquid film on unsteady stretching sheet. Siddiqui et al. (2007) presented the thin film flow of two non-Newtonian fluids namely, Sisko and an Oldroyd 6-constant fluid on a vertical moving belt. Hayat et al. (2008) presented an exact solution for the thin film flow problem of a third grade on an inclined plane. The problem of the flow and heat transfer in a thin film of power-law fluid on an unsteady stretching surface has been investigated by Chen (2003, 2006) where he also studied the effect of viscous dissipation on heat transfer in a non-Newtonian thin liquid film over an unsteady stretching sheet. The flow and heat transfer problem of a second grade fluid film over an unsteady stretching sheet has been presented by Hayat et al. (2008). Abel et al. (2009) investigated the effect of non-uniform heat source on MHD heat transfer in a liquid film over an unsteady stretching sheet. Sajid et al. (2009) presented exact solutions for thin film flows of a micropolar fluid down an inclined plane on moving belt and down a vertical cylinder. Mahmoud and Megahed (2009) investigated the effects of variable viscosity and thermal conductivity on the flow and heat transfer of an electrically conducting non-Newtonian power-law fluid within a thin liquid film over an unsteady stretching sheet in the presence of a transverse magnetic field.

Non of the above authors deals with the problem involving the thermal radiation on the flow and heat transfer in a liquid film on unsteady stretching surface. Thermal radiation effects may play an important role in controlling heat transfer in industry where the desired product with a sought characteristics depends on the heat controlling factors to some extent. The effect of thermal radiation on the flow and heat transfer of a non-Newtonian fluids has been studied by several authors (Aliakbar et al. 2009; Mahmoud 2007; Raptis 1998, 1999; Siddheshwar and Mahabaleswar 2005; Hayat and Qasim 2010). The transfer of heat due to the missing electromagnetic waves (thermal radiation) has been presented by Baleanu et al. (2015). Available literature shows that the effect of thermal radiation on Maxwell liquid film over an unsteady stretching sheet immersed in a porous medium is not being carried out. Therefore, the aim of this study is to investigate the influence of thermal radiation on heat transfer in an upper-convected Maxwell liquid film over an unsteady stretching surface embedded in a porous medium.

## Formulation of the problem

**Fig. 1:**
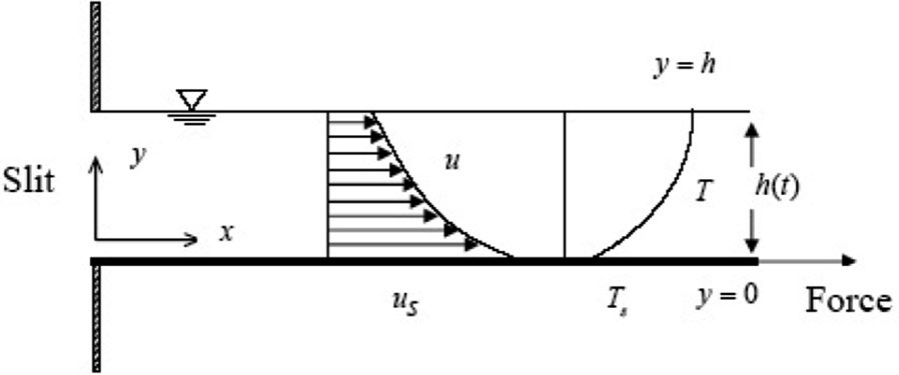
Schematic of the physical system

Consider a laminar and incompressible unsteady flow of an upper-convected Maxwell fluid in a thin liquid film on a stretching surface immersed in a porous medium issuing from a narrow slit at the origin as shown in Fig. 1. The continuous surface aligned with the *x* axis at $y=0$ moves in its own plane with a velocity $u_{s}(x,t)$ and temperature distribution $T_{s}(x,t)$. A thin liquid film of uniform thickness *h*(*t*) lies on the horizontal surface.

The basic equations for mass, momentum and energy in the thin liquid layer using the usual boundary layer approximations are:

1 $$\begin{aligned}&\frac{\partial u}{\partial x}+\frac{\partial v}{\partial y}=0, \end{aligned}$$

2 $$\begin{aligned}&\frac{\partial u}{\partial t}+u\frac{\partial u}{\partial x}+v\frac{\partial u}{\partial y}=\frac{\mu }{\rho }\frac{\partial ^{2} u}{\partial y^{2}}-\lambda _{1}\left[ u^{2}\frac{\partial ^{2} u}{\partial x^{2}}+v^{2}\frac{\partial ^{2} u}{\partial y^{2}}+2uv\frac{\partial ^{2} u}{\partial x\partial y}\right] -\frac{\mu }{\rho k}u, \end{aligned}$$

3 $$\begin{aligned}&\frac{\partial T}{\partial t}+u\frac{\partial T}{\partial x}+v\frac{\partial T}{\partial y}=\frac{\kappa }{\rho c_{p}} \frac{\partial ^{2} T}{\partial y^{2}}-\frac{1}{\rho c_{p}}\frac{\partial q_{r}}{\partial y}, \end{aligned}$$

where *u* and *v* are the velocity components along the *x* and *y* directions, respectively. $\rho$ is the fluid density, *T* is the temperature of the fluid, *t* is the time, $\mu$ is the viscosity of the fluid, $\lambda _{1}(t)=\lambda _{0}(1-at)$ is the relaxation time of period, $\lambda _{0}$ is a constant (Mukhopadhyay 2012), *k* is the permeability of the porous medium, $\kappa$ is the thermal conductivity, $q_{r}$ is the radiative heat flux and $c_{p}$ is the specific heat at constant pressure.

The appropriate boundary conditions for the present problem are:

4 $$\begin{aligned} u = u_{s}, v=0, \quad T=T_{s} \quad at \quad y=0, \end{aligned}$$

5 $$\begin{aligned} \frac{\partial u}{\partial y}=\frac{\partial T}{\partial y}=0 \quad at \quad y=h, \end{aligned}$$

6 $$\begin{aligned} v= \frac{d h}{d t} \quad at \quad y=h, \end{aligned}$$

where *h* is the thickness of the liquid film which has been assumed to be uniform. The flow is caused by stretching the elastic surface at $y=0$ such that the continuous sheet moves in the $x$-direction with the velocity (Dandapat et al. 2003):

7 $$\begin{aligned} u_{s}=\frac{bx}{(1-at)}, \end{aligned}$$

where *a* and *b* are positive constants with dimension $(time)^{-1}$. Eqation (5) imposes a kinematic constraint of the fluid motion. $T_{s}$ is the surface temperature of the stretching sheet, which varies with the distance *x* along the sheet and time *t* in the form (Dandapat et al. 2003):

8 $$\begin{aligned} T_{s}=T_{0}-T_{ref}\left[ \frac{bx^{2}}{2\nu }\right] (1-at)^{\frac{-3}{2}}, \end{aligned}$$

here $T_{0}$ is the temperature at the slit and $T_{ref}$ is the reference temperature, which can be taken either as a constant reference temperature or a constant temperature difference. In the present work $T_{ref}$ will be taken as $T_{ref}=T_{0}$. It should be noticed that the Eqs. (7) and (8), on which the following analysis is based, are valid only for time $t<\frac{1}{a}$.

The radiative heat flux $q_{r}$ is employed according to Rosseland approximation (Raptis 1999) such that:

9 $$\begin{aligned} q_{r}=-\frac{4\sigma ^{*}}{3k^{*}}\frac{\partial T^{4}}{\partial y}, \end{aligned}$$

where $\sigma ^{*}$ is the Stefan-Boltzmann constant and $k^{*}$ is the mean absorption coefficient. Following, Raptis (1998), we assume that the temperature difference within the flow are small such that $T^{4}$ may be expressed as a linear function of the temperature. Expanding $T^{4}$ in a Taylor series about $T_{0}$ and neglecting higher-order terms, we have:

10 $$\begin{aligned} T^{4}\cong 4T_{0}^{3}T-3T_{0}^{4}. \end{aligned}$$

We introduce the following dimensionless variables (Dandapat et al. 2003):

11 $$\begin{aligned} \eta= \left( \frac{b}{\nu }\right) ^{\frac{1}{2}}(1-at)^{\frac{-1}{2}}y, \end{aligned}$$

12 $$\begin{aligned} \psi= \left[ \nu b (1-at)^{-1}\right] ^{\frac{1}{2}}xf, \end{aligned}$$

13 $$\begin{aligned} T= T_{0}-T_{ref}\left[ \frac{bx^{2}}{2\nu }\right] (1-at)^{\frac{-3}{2}}\theta , \end{aligned}$$

where $\psi$ is the stream function that satisfies the continuity Eq. (1). The velocity components are:

14 $$u=\frac{\partial \psi}{\partial y}= \left (\frac{b
x}{1-at} \right)f^{'}(\eta), v=-\frac{\partial \psi}{\partial
x}=-\left(\frac{\nu b}{1-at}\right)^{\frac{1}{2}}f(\eta).$$

By using the transformation given in Eqs. (11)–(14), the governing Eqs. (2)–(3) and the boundary conditions (4–6) become:

15 $$\begin{aligned}&f^{'''}+ff^{''}-f^{'2}-S\left( f^{'}+\frac{\eta }{2}f^{''}\right) -De\left[ f^{2}f^{'''}-2ff^{'}f^{''}\right] -Df^{'}=0, \end{aligned}$$

16 $$\begin{aligned}&\frac{1}{Pr}[(1+R)\theta ^{''}]+f\theta ^{'}-2f^{'}\theta -\frac{S}{2}\left[ 3\theta +\eta \theta ^{'}\right] =0,\end{aligned}$$

17 $$\begin{aligned}&f=0, \quad f^{'}=1, \quad \theta =1 \quad at \quad \eta =0, \end{aligned}$$

18 $$\begin{aligned}&f^{''}=0, \quad \theta ^{'}=0 \quad at \quad \eta =\beta , \end{aligned}$$

19 $$\begin{aligned}&f=\frac{1}{2}S\beta \quad at \quad \eta =\beta , \end{aligned}$$

where a prime denotes differentiation with respect to $\eta$ , $S=\frac{a}{b}$ is the unsteadiness parameter, $Pr=\frac{\mu c_{p}}{\kappa }$ is the Prandtl number, $R=\frac{16 \sigma ^{*}T_{0}^{3}}{3k^{*}\kappa }$ is the radiation parameter, $De=\frac{\lambda _{1}(t) b}{(1-at)}$ is the *local* Deborah number, $D=\frac{1}{Da}$ is the local Darcy parameter and $Da=\frac{\rho b k(t)}{\mu (1-at)}$ is the Darcy number. For similarity solution the permeability *k* is taken in the form $k(t)=(1-at)$. $\beta$ is unknown constant, denotes the value of $\eta$ at the free surface, which must be determined as a part of the present problem. It is noticed that although the dimensionless film thickness $\beta$ is constant which depends only on *S*, the actual film thickness *h*(*t*) depends only on time *t*, then the actual film thickness $\beta =\eta$ at $y=h$ i.e.

20 $$\begin{aligned} h(t)=\beta \left( \frac{b}{\nu }\right) ^{\frac{-1}{2}}(1-at)^{\frac{1}{2}}. \end{aligned}$$

The physical quantities of interest are the local skin-friction coefficient $Cf_{x}$ and the local Nusselt number $Nu_{x}$ which are defined as:

21 $$\begin{aligned} Cf_{x}= -\frac{2\mu \left( \frac{\partial u}{\partial y}\right) _{y=0}}{\rho u_{s}^{2}}=-2Re_{x}^{\frac{-1}{2}}f^{''}(0), \end{aligned}$$

22 $$\begin{aligned} Nu_{x}= -\frac{x\left( \frac{\partial T}{\partial y}\right) _{y=0}}{ (T_{s}-T_{0})}=-Re_{x}^{\frac{1}{2}}\theta ^{'}(0), \end{aligned}$$

where $Re_{x}=\frac{u_{s}x}{\nu }$ is the local Reynolds number.

## Results and discussion

The exact analytical solution for the system of non-linear ordinary differential Eqs. (15) and (16) with the boundary conditions (17)–(19) is not feasible. Therefore Eqs. (14) and (15) along with the boundary conditions (16)–(18) were solved numerical using the fourth-order Runge-Kutta integration scheme with the shooting method. In order to validate of the numerical method, we have compared the values of $f^{''}(0)$, $\theta (\beta )$ and $-\theta ^{'}(0)$(in the absence of *De*, *D* and *R* ) with those obtained by Abel et al. (2009) (in the absence of *M* and *Ec*) and found in good agreement as shown in Tables 1 and 2.

**Table 1:** Comparison of $\beta$ and $-f^{''}$(0) with $De=D=0$

*S*	Abel et al. (2009)		Present result	
	$\beta$	$-f^{''}$(0)	$\beta$	$-f^{''}$(0)
0.4	4.981455	1.134098	4.981455	1.134096
0.6	3.131710	1.195128	3.131711	1.195125
0.8	2.151990	1.245805	2.151992	1.245805
1.0	1.543617	1.277769	1.543616	1.277769
1.2	1.127780	1.279171	1.127781	1.279171
1.4	0.821033	1.233545	0.821032	1.233545
1.6	0.576176	1.114941	0.576175	1.114939
1.8	0.356390	0.867416	0.356389	0.867416

**Table 2:** Comparison of $\theta (\beta )$ and $-\theta ^{'}$(0) with $De=D=R=0$

*Pr*	*S*	$\beta$	Abel et al. (2009)		Present result	
			$\theta (\beta )$	$-\theta ^{'}$(0)	$\theta (\beta )$	$-\theta ^{'}$(0)
0.01	0.8	2.151990	0.960438	0.042120	0.960480	0.042042
0.1	0.8	2.151990	0.692269	0.351920	0.692533	0.351377
1.0	0.8	2.151990	0.097825	1.671919	0.097884	1.671003
2.0	0.8	2.151990	0.024869	2.443914	0.024862	2.443866
3.0	0.8	2.151990	0.008324	3.034915	0.008311	3.036115
0.01	1.2	1.127780	0.982312	0.033515	0.982331	0.033459
0.1	1.2	1.127780	0.843485	0.305409	0.843622	0.304963
1.0	1.2	1.127780	2.86634	1.773772	2.86718	1.773030
2.0	1.2	1.127780	0.128174	2.638431	0.128123	2.638725
3.0	1.2	1.127780	0.067737	3.280329	0.067645	3.281949

**Fig. 2:**
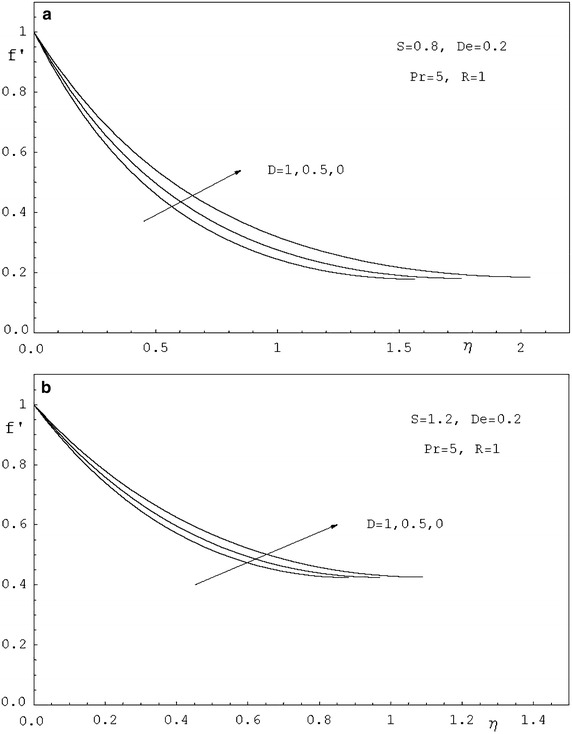
**a** Velocity profiles for various values of *D*. **b** Velocity profiles for various values of *D*

**Fig. 3:**
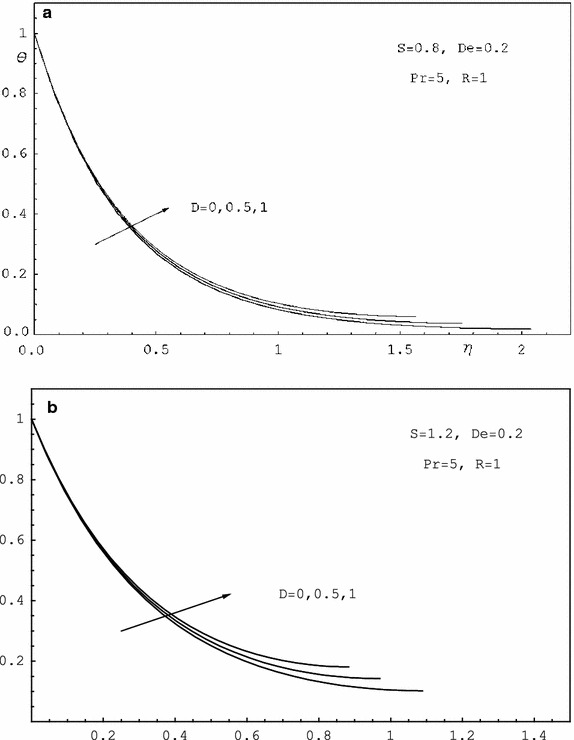
**a** Temperature profiles for various values of *D*.** b** Temperature profiles for various values of *D*

**Fig. 4:**
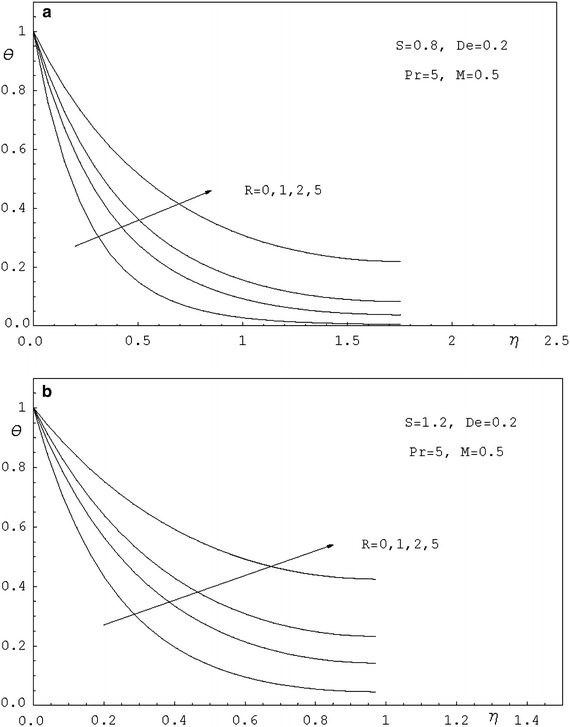
**a** Temperature profiles for various values of *R*. **b** Temperature profiles for various values of *R*

**Fig. 5:**
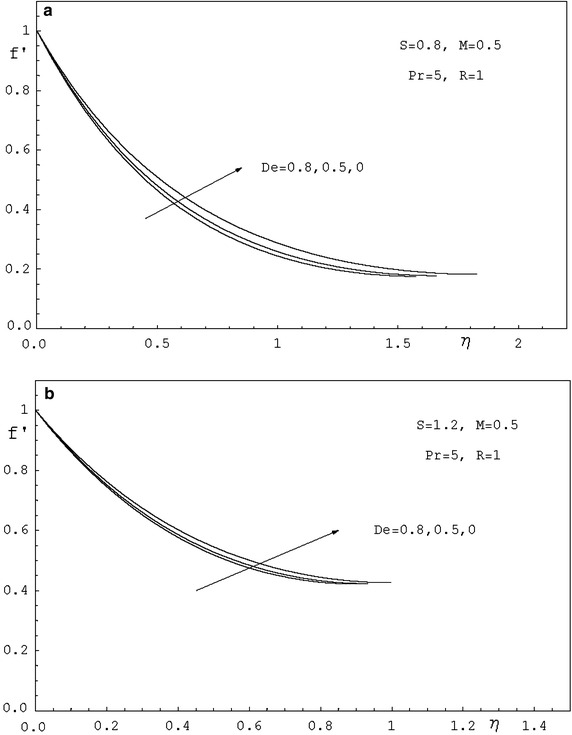
**a** Velocity profiles for various values of *De.* **b** Velocity profiles for various values of *De*

**Fig. 6:**
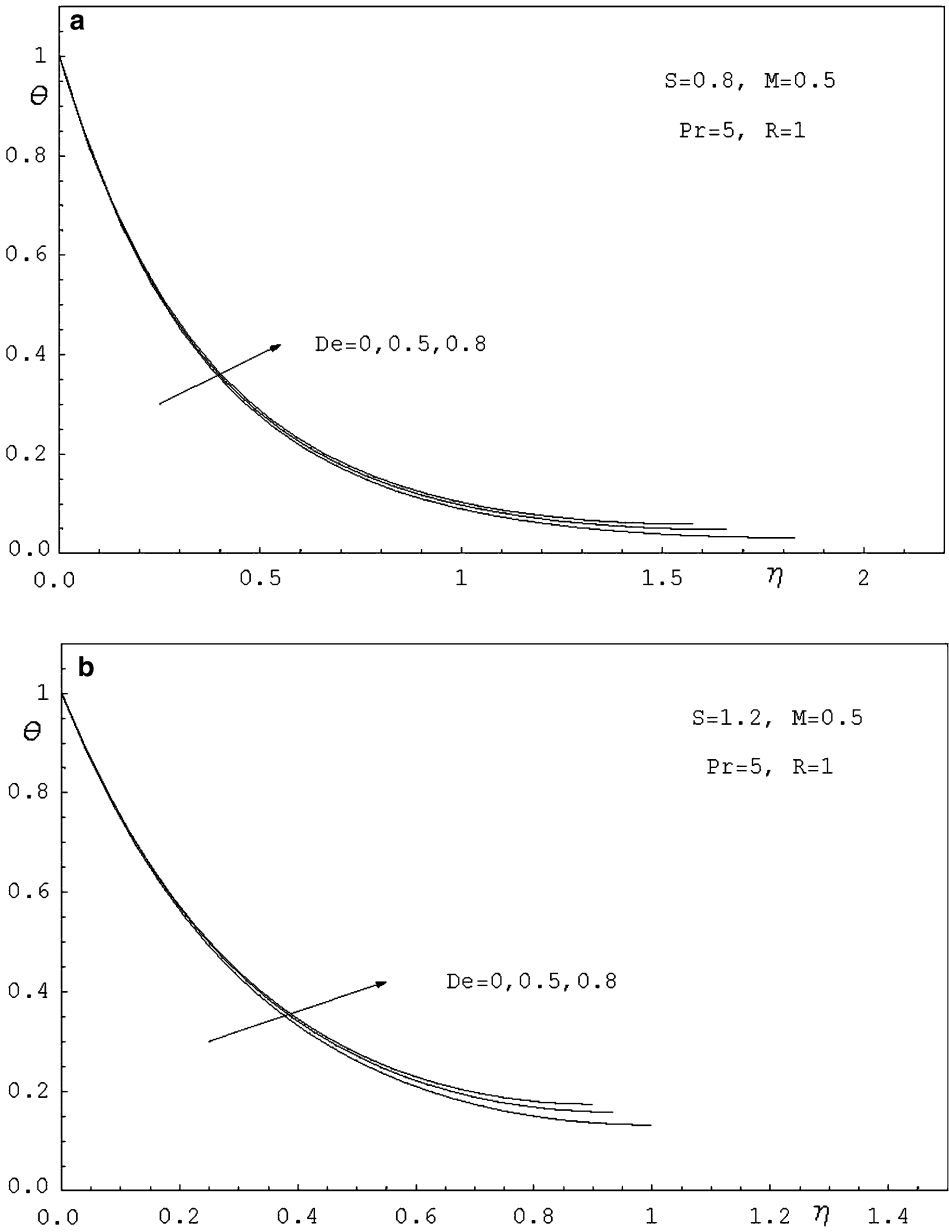
**a** Temperature profiles for various values of *De.* **b** Temperature profiles for various values of *De*

**Fig. 7:**
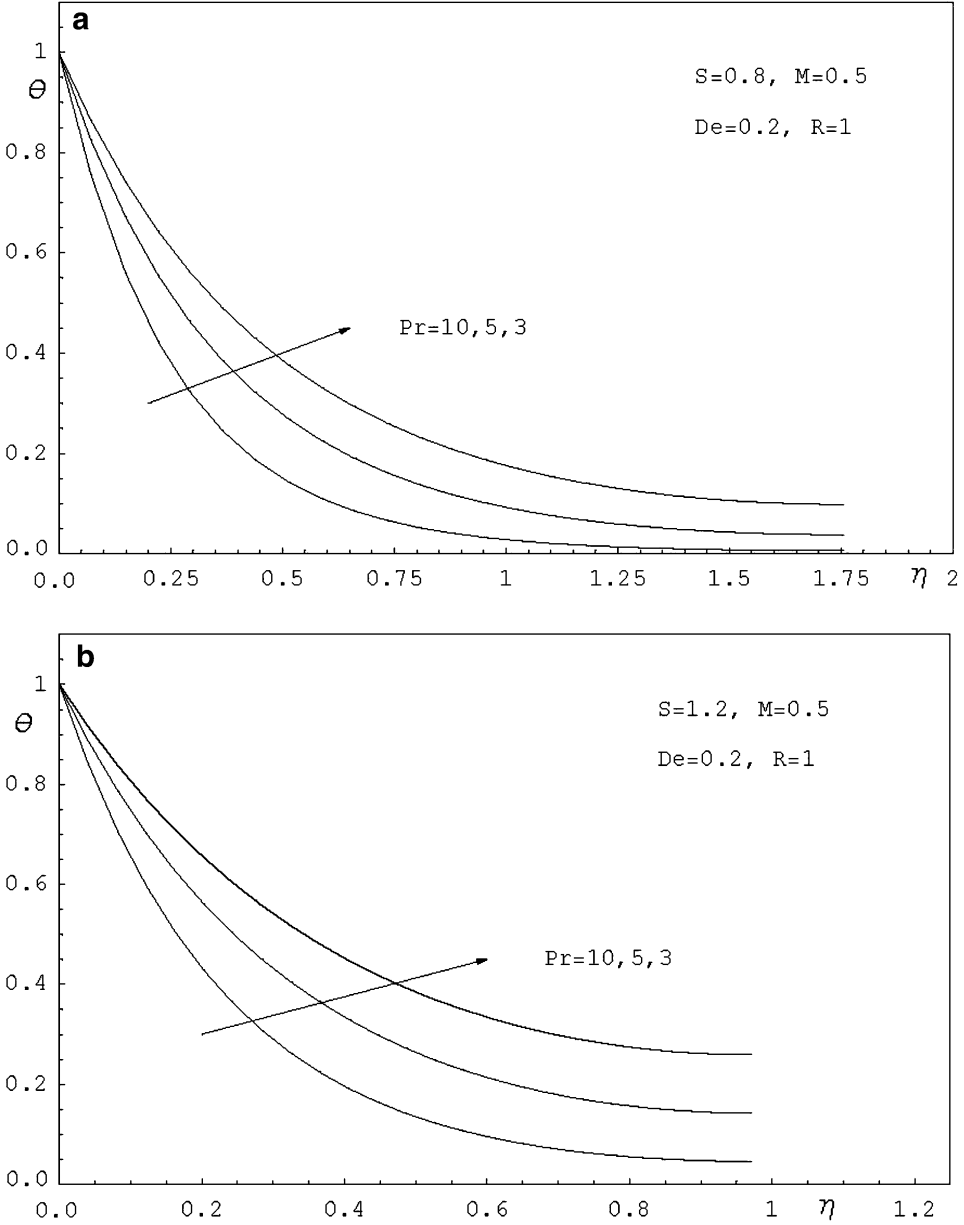
**a** Temperature profiles for various values of *Pr.* **b** Temperature profiles for various values of *Pr*

To study the effects of various parameters like the radiation parameter *R*, the Darcy parameter *D*, the Deborah number *De* and the Prandtl number *Pr* on the dimensionless velocity $f^{'}(\eta )$ and the dimensionless temperature $\theta (\eta )$, numerical calculations have been carried out for different values of *R*, *D*, *De* and *Pr* as shown in Figs. 2, 3, 4, 5, 6 and 7. Also, the variation of the local skin-friction coefficient and the local Nusselt number with the change in the parameters *R*, *D*, *De* and *Pr* are illustrated in Table 3. Figure 2 demonstrates the effect of the Darcy parameter *D* on the horizontal velocity profiles for different values of *S*. It is revealed that the transverse velocity decreases as the Darcy parameter increases. Also, it is noticed that the film thickness $\beta$ decreases as the unsteadiness parameter *S* increases. The dimensionless temperature profiles $\theta (\eta )$ are depicted in Fig. 3 for various values of *D*. It is observed that the temperature at a point increases with increase in *D*. This is due to the fact that the porous medium produces a resistive type of force which causes a reduction in the fluid velocity and enhancing the temperature. The effect of the radiation parameter on the dimensionless temperature $\theta (\eta )$ is displayed in Fig. 4. It is seen that the increase of the radiation parameter leads to an increase in the temperature at any point. This is because the increase in the radiation parameter implies higher surface heat flux and thereby increasing the temperature of the fluid. The influence of the Deborah number on the transverse velocity $f^{'}(\eta )$ is shown in Fig. 5. It is shown that at any point $f^{'}(\eta )$ decreases as *De* increases. Also, it is seen that the transverse velocity decreases with $\eta$ and the film thickness decreases with increasing *De*. Figure 6 demonstrates that at any point the dimensionless temperature $\theta (\eta )$ increases with the increasing of the Deborah number *De*. Also, it is noticed that the dimensionless temperature decreases with $\eta$. The variation of the dimensionless temperature against $\eta$ for various values of the Prandtl number are displayed in Fig. 7. It is found that the temperature decreases with $\eta$ until its value at the free surface. It is also, observed that the temperature decreases with the increase of the Prandtl number. This is due to the fact that a fluid with larger Prandtl number possesses larger heat capacity, and hence augments the heat transfer.

**Table 3:** Values of $-f^{''}$(0) and $-\theta ^{'}$(0) for various values of *D*, *R*, *De*, *S* and *Pr*

*D*	*R*	*De*	*Pr*	*S*	$-f^{''}$(0)	$\theta (\beta )$	$-\theta ^{'}$(0)
0.0	1	0.2	5	0.8	1.2827	0.0187	2.7469
0.5	1	0.2	5	0.8	1.4606	0.0378	2.7177
1	1	0.2	5	0.8	1.6187	0.0600	2.6910
0.0	1	0.2	5	1.2	1.3056	0.1023	2.9708
0.5	1	0.2	5	1.2	1.4548	0.1427	2.9404
1	1	0.2	5	1.2	1.5901	0.1813	2.9087
0.5	0	0.2	5	0.8	1.4606	0.0059	3.9397
0.5	1	0.2	5	0.8	1.4606	0.0378	2.7177
0.5	2	0.2	5	0.8	1.4606	0.0826	2.1753
0.5	5	0.2	5	0.8	1.4606	0.2187	1.4581
0.5	0	0.2	5	1.2	1.4548	0.0458	4.2638
0.5	1	0.2	5	1.2	1.4548	0.1426	2.9404
0.5	2	0.2	5	1.2	1.4548	0.2323	2.3364
0.5	5	0.2	5	1.2	1.4548	0.4247	1.5150
0.5	1	0.0	5	0.8	1.4280	0.0316	2.7249
0.5	1	0.2	5	0.8	1.4606	0.0378	2.7177
0.5	1	0.5	5	0.8	1.5088	0.0479	2.7067
0.5	1	0.0	5	1.2	1.4311	0.1322	2.9478
0.5	1	0.2	5	1.2	1.4548	0.1426	2.9404
0.5	1	0.5	5	1.2	1.4904	0.1584	2.9286
0.5	1	0.2	3	0.8	1.4606	0.0984	2.0508
0.5	1	0.2	5	0.8	1.4606	0.0378	2.7177
0.5	1	0.2	10	0.8	1.4606	0.0059	3.9397
0.5	1	0.2	3	1.2	1.4548	0.2591	2.1955
0.5	1	0.2	5	1.2	1.4548	0.1427	2.9404
0.5	1	0.2	10	1.2	1.4548	0.0458	4.2638

The numerical values of the local skin-friction and the local Nusselt number in terms of $-\theta ^{'}(0)$ for various values of the Darcy parameter *D*, the radiation parameter *R*, the Deborah number *De* and the Prandtl number *Pr* for both cases $S=0.8$ and $S=1.2$ are tabulated in Table 3. It can be seen that the local skin-friction coefficient increased by increasing *D* or *De*, whereas the local Nusselt number decreases with the increasing the Darcy parameter and the Deborah number. Also, it is noticed that the local Nusselt number decreases as the radiation parameter increases and increases with the increase of the Prandtl number. However it is found that the local Nusselt number decreases as *S* increases whereas the local skin-friction coefficient decreases with the unsteadiness parameter. Moreover, it is observed that the free surface temperature $\theta (\beta )$ increases by increasing *D*, *R*, *S* and *De* whereas it decreased with increasing the Prandtl number.

## Conclusions

A theoretical analysis is performed to study thermal radiation effects on flow and heat transfer in an upper convected Maxwell liquid film on an unsteady stretching sheet embedded in a porous medium. The governing equations are transformed to a system of non-linear ordinary differential equations which is solved numerically using the fourth order Runge-Kutta scheme with the shooting technique. The main conclusions which have been found from the present study are:

1. The velocity and the film thickness decreases with increasing the Darcy parameter and the Deborah number.
2. Increasing values of the Darcy parameter, radiation parameter and Deborah number leads to an increase in the temperature.
3. The temperature decreases with increasing the Prandtl number.
4. The Darcy parameter and the Deborah number have the effect of enhancing the local skin-friction coefficient.
5. The local Nusselt number decreases by increasing the radiation parameter, the Darcy parameter and the Deborah number and increases with increasing the Prandtl number.
6. The free surface temperature decreases as the Darcy parameter, the radiation parameter and the Deborah number increase while it decreases as the Prandtl number increases.
